# Nutrition, Behavior Change and Physical Activity Outcomes From the PEARS RCT—An mHealth-Supported, Lifestyle Intervention Among Pregnant Women With Overweight and Obesity

**DOI:** 10.3389/fendo.2019.00938

**Published:** 2020-02-04

**Authors:** Kate M. Ainscough, Eileen C. O'Brien, Karen L. Lindsay, Maria A. Kennelly, Elizabeth J. O'Sullivan, Orna A. O'Brien, Mary McCarthy, Giuseppe De Vito, Fionnuala M. McAuliffe

**Affiliations:** ^1^UCD Perinatal Research Centre, School of Medicine, National Maternity Hospital, University College Dublin, Dublin, Ireland; ^2^School of Biological and Health Sciences, Technological University Dublin, Dublin, Ireland; ^3^Food Business and Development, University College Cork, Cork, Ireland; ^4^UCD Institute of Sport and Health, School of Public Health, Physiotherapy and Sports Science, University College Dublin, Dublin, Ireland

**Keywords:** mHealth, pregnancy, behavior change, maternal diet, lifestyle intervention, overweight and obese pregnancy

## Abstract

**Background:** Diet quality and physical activity positively impact pregnancy outcomes among women with obesity, but successful lifestyle interventions require intense clinician time. We aimed to investigate the impact of a behavioral-lifestyle intervention (PEARS) supported by a smartphone app among pregnant women with overweight and obesity, on nutrient intake, behavioral stage-of-change and physical activity.

**Methods:** Pregnant women (BMI 25–39.9 kg/m^2^, measured, *n* = 565) were randomized at 15.6 weeks' gestation to the intervention (*n* = 278), or a control group (*n* = 287) (ISRCTN29316280). The intervention was grounded in behavior-change theory. Participants received nutrition (low glycaemic index and healthy eating) and exercise advice, a smartphone app and fortnightly emails. The control group received usual care which does not include dietary advice. At baseline and 28 weeks' gestation, dietary data were obtained through 3-day food diaries (*n* = 290 matched), and stage-of-change and physical activity data were self-reported. App usage data were collected.

**Results:** There were no differences between the groups at baseline. Compared with the control group, the intervention group had improved dietary intakes post-intervention with; lower glycaemic index (MD −1.75); free sugars (%TE) (MD −0.98); fat (%TE) (MD −1.80); and sodium (mg) (MD −183.49). Physical activity (MET-minutes/week) was higher in the intervention group post-intervention (MD 141.4; 95% CI 62.9, 219.9). The proportion of participants at “maintenance” stage-of-change for physical activity was higher in the intervention group (56.3 vs. 31.2%). App use was associated with lower glycaemic index and less energy from free sugars, but not with physical activity.

**Conclusion:** A behavioral-lifestyle intervention in pregnancy supported by a smartphone app improved dietary intakes, physical activity, and motivation to engage in exercise.

## Introduction

Women with raised body mass index (BMI) in early pregnancy have an increased risk of gestational diabetes mellitus (GDM), excess gestational weight gain (GWG), and infants born large-for-gestational-age (LGA) (1). Improving dietary and physical activity behaviors could help to negate such outcomes (2, 3), and could assist in addressing the global health concern of maternal obesity. While pregnancy is a unique period of change when women may be motivated to make healthier lifestyle choices to benefit the health of their baby (4, 5), women with a higher BMI may perceive more barriers and have lower self-efficacy to successfully implement diet and exercise behavior changes during pregnancy (5).

Evidence from previous studies shows that higher pre-pregnancy BMI is associated with lower dietary quality (6), and dietary quality may decline with advancing gestation in pregnant women with obesity (7). Furthermore, it has been reported that as few as 21% of all women meet recommendations for physical activity during pregnancy (8, 9).

Some behavioral lifestyle interventions have improved physical activity and dietary behaviors among pregnant women with raised BMI (2, 3, 10, 11), with subsequent reductions in GWG (2, 3, 10), infant birthweight (2, 11) and improvements in maternal glucose homeostasis (2, 11). However, these interventions are typically intense in frequency and content, involving multiple face-to-face sessions, which may be difficult and expensive to implement in routine clinical settings.

Mobile-Health (mHealth) technologies offer the potential to support traditional lifestyle interventions without increasing contact hours with clinicians (12). Smartphone apps have been proposed as a novel method to provide antenatal education, for example in improving glycaemic control (13), gestational weight gain (14, 15) and maternal self-efficacy and mental well-being (16). A recent meta-analysis demonstrated the increasing prevalence of mHealth-supported randomized controlled trials for weight management in pregnancy (17). Despite a greater publication of studies using mHealth tools to promote maternal behaviors, few have focused on diet and physical activity specifically, and among those that have, mixed results have been reported (18, 19). Freely available smartphone apps are generally low quality and contain limited behavior change techniques and pregnancy-specific nutrition information; thus, it has been suggested that they are not an appropriate resource for pregnant women (20, 21). There is a need for pregnancy-specific smartphone apps that deliver high quality, evidenced-based nutritional and physical activity advice, with demonstrated effectiveness that can be trusted by healthcare professionals and users.

We have previously found that the low glycaemic index diet in pregnancy (ROLO study) is a successful dietary approach to improving maternal and fetal outcomes (22). Using our experience, we built on the ROLO randomized controlled trial, adding physical activity and mHealth components, and embedded with behavior change approaches.

The aim of this study is to investigate the impact of the PEARS behavioral lifestyle intervention, supported by a smartphone app, on maternal dietary intakes, behavioral stage-of-change and physical activity. This paper also describes the level of engagement with the study-specific app and explores dietary and physical activity outcomes according to app usage.

## Materials and Methods

### Study Design and Setting

The PEARS (Pregnancy Exercise And nutrition Research Study) trial was carried out at The National Maternity Hospital, Dublin, Ireland. This was a randomized controlled trial of a diet and exercise lifestyle intervention with smartphone application support to prevent GDM (ISRCTN29316280). The study received ethical approval from The National Maternity Hospital Ethics Committee in October 2012. Between 2013 and 2016, 565 women were recruited at their first antenatal visit. Eligibility criteria included: 10–18 weeks' gestation, singleton pregnancy, 18–45 years of age, BMI ≥25 kg/m^2^ and ≤39.9 kg/m^2^, and in possession of a smartphone. Exclusion criteria were: previous GDM and any medical condition requiring treatment. Information about the study was given and written informed consent was obtained from all women. Participants were then randomized in a 1:1 ratio to the intervention or control group. A biostatistician created a computer-generated random sequence, stratified by BMI category. Allocation was concealed in sequentially-numbered, sealed, opaque envelopes. Following recruitment at the initial antenatal visit to the hospital, women returned for their first study visit approximately 2 weeks later (mean gestation of 16 weeks). The randomization envelope was opened at the participant's first study visit to reveal group allocation. Women randomized to the control group received standard antenatal care which does not include formal dietary or physical activity advice.

Details of the PEARS trial and primary outcome results have been published (23, 24). In brief, the incidence of GDM did not differ between those in the intervention and control arm; however, those in the intervention arm significantly reduced their glycaemic load and increased their exercise intensity (24).

### Lifestyle Intervention

The intervention was grounded in Control Theory and Social Cognitive Theory. These theories were subsequently mapped to supporting behavior-change techniques (BCTs) used to deliver intervention content. Published taxonomies of BCTs were used (25–27). Terminology from the CALO-RE Taxonomy of BCTs was used primarily (26), incorporating some BCTs from Taxonomy V1 (27). The Behavior Change Wheel, in which the COM-B model centers (26) was used to map out and describe how the BCTs functioned within the intervention to increase self-efficacy for behavior-change. The COM-B model describes self-efficacy as three behavioral constructs; Capability, Opportunity, and Motivation for Behavior-change (26).

The intervention was delivered by the research nutritionist/dietitian and obstetrician. Dietary advice centered on reducing the GI and glycaemic load (GL) of foods consumed, in addition to general healthy eating advice for pregnancy, including the food pyramid and energy recommendations during each trimester of pregnancy. Individualized low-GI dietary goals were set using the SMART Goals (goals must be specific, measureable, achievable, realistic, and have a time component) principle (28). Physical activity was prescribed based on the American Congress of Obstetricians and Gynecologists (ACOG) recommendations (9) of moderate intensity for at least 30 min, 5–7 days per week, to achieve 150 min per week (9). Individual physical activity goals were set. Institute of Medicine (IOM) guidelines for recommended GWG (29) were discussed and participants were advised regarding appropriate GWG.

**Figure 1:**
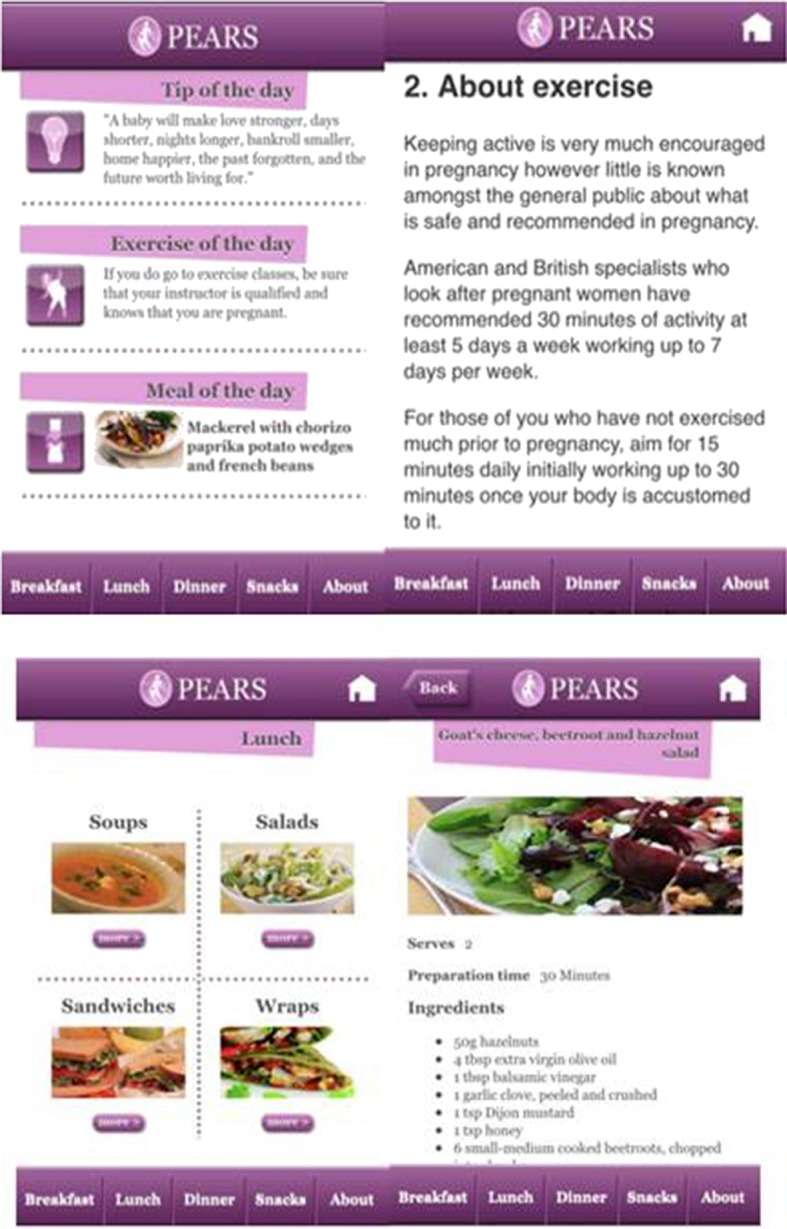
Screenshot samples from the PEARs Study smartphone app.

Women in the intervention arm were also provided with access to the study-specific smartphone app. Daily app usage was encouraged. The key feature of the app was a database of low-GI recipes for breakfast, lunch, dinner, and snacks. The “Home” page alternated an exercise of the day, a link to a meal of the day and a tip of the day (motivational quote or pregnancy advice). Brief information on physical activity and the PEARS trial was also contained in the app. Examples of the app interface and content are shown in Figure 1.

For the duration of the intervention, participants received fortnightly follow-up emails to review their individualized goals and encourage compliance. At 28- and 34-weeks' gestation, the research team had brief, face-to-face contact with participants.

### Dietary Intakes, Physical Activity and Behavioral Stage-of-Change Assessment

At the baseline (initial recruitment visit) and 28 weeks' gestation study visits, participants were instructed to complete lifestyle questionnaires and to record all food and beverages in 3-day food diaries. Instructions were provided to record all food and beverages consumed for 3 days; 2 week days and 1 weekend day to assess normal eating habits. The types and amount of food consumed was recorded by participants in household measures (e.g., teaspoons, tablespoons) or using the weight listed on food and beverage packaging. Data were entered to Nutritics Professional Nutrition Analysis Software, version 4.267, Research Edition (Nutritics, Dublin, Ireland, www.nutritics.com). Participant's mean daily nutrient intakes, including macronutrients as percentages of total energy were calculated for pre- and post-intervention. Mean daily GI was determined using the formula [GL/Carbohydrate (g) × 100] (30).

Energy under-reporting was assessed using the Goldberg method (31), which has been previously used in pregnancy (30). With this method, each participant's basal metabolic rate (BMR) was calculated using Henry Oxford Equations (32), and the ratio of energy intake (EI) to BMR (EI:BMR) was calculated. A Goldberg ratio of ≤0.9 was used to define under-reporting. It was decided to run analyses both with and without under-reporters.

Physical activity was reported using a questionnaire adapted from the SLÁN 2002 survey (33) and previously validated in pregnancy (8). Frequency of 30 min intervals of light, moderate and vigorous leisure time activity per week were recorded and metabolic equivalents of task minutes (MET-mins) per week, were calculated (34). Behavioral stage-of-change was measured to examine women's perceptions of their readiness to engage in physical activity behaviors, using a questionnaire previously validated in a pregnant and non-pregnant cohort (35, 36). The model contains five stages of readiness to engage in behaviors; pre-contemplation, contemplation, preparation, action, and maintenance (Supplementary Material).

### App Usage Assessment

The app tracked participants' total usage and the number of times pages were accessed. Frequency of use was assessed for six app pages; Home, About, Breakfast, Lunch, Dinner, and Snacks. App usage was characterized in two ways; (i) app-users vs. non-app-users (categorical): App-users were defined as those who used the app at least once after the initial use on the day of download, while non-app-users never used the app, or their only recorded use was the first time it was opened after download; (ii) instances of app use (continuous): One instance of use was defined as one 15-min period of access to the app.

### Maternal Characteristics

Weight was recorded at baseline, 28- and 34-week study visits. Height was recorded at baseline and BMI (kg/m^2^) calculated using early pregnancy weight. Maternal age, gestational age at baseline, parity, ethnicity, education level, smoking status, and supplement use were collected from medical charts and self-reported in the lifestyle questionnaire. Neighborhood deprivation data were obtained through the Pobal Haase-Pratschke Deprivation Index address-mapping tool (37).

### Statistical Analyses

Variables were visually assessed for normality using histograms. Independent-samples *t*-tests were used for continuous variables and Chi-Squared tests for categorical variable. Analysis of covariance was performed to assess between-group differences at 28 weeks' gestation, controlling for baseline values as per the European Medicines Agency guidelines for clinical trials (38). Linear regression analysis was used to assess odds ratio for meeting the ACOG physical-activity guidelines. Linear regression analysis was used to assess the association between app usage instances with nutrient intakes (intervention group participants only). All statistical analyses were performed on IBM SPSS software for Windows version 24.0 (SPSS Inc, Chicago, IL).

## Results

### Study Participants

**Figure 2:**
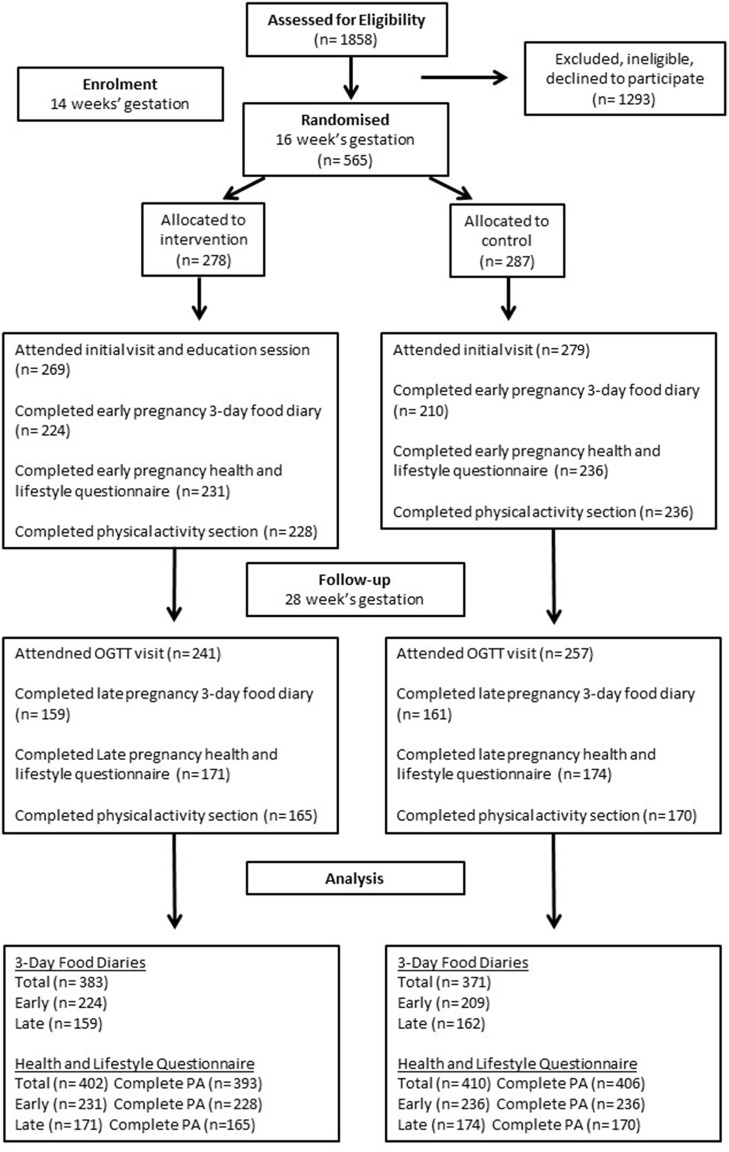
CONSORT flow diagram of study participants.

Figure 2 illustrates the flow of subjects through the study. In total, 1,858 women were approached for eligibility; of these, 565 women agreed to participate, provided consent and were randomized to the intervention or control group, whilst 1,293 were ineligible, declined to participate or changed their mind regarding participation prior to randomization. Apart from education level, which was significantly higher in the control group, there were no differences in participant characteristics between intervention and control groups at baseline (Table 1).

**Table 1:** PEARs participant baseline characteristics (*n* = 565)^a^^,^^b^.

	**Intervention**		**Control**		***P***
	***n***	**Mean ± SD**	***n***	**Mean ± SD**	
Age (years)	271	32.84 ± 4.60	283	32.22 ± 4.23	0.100
Height (cm)	278	164.3 ± 6.46	287	164.6 ± 6.73	0.597
Early-pregnancy weight (kg)	278	79.57 ± 11.35	287	78.87 ± 11.03	0.460
Early-pregnancy BMI (kg/m^2^)	278	29.44 ± 3.60	287	29.07 ± 3.28	0.196
25–29.9 kg/m^2^ (overweight) [*n* (%)]	278	184 (66.2)	287	195 (67.9)	0.723
30–39.9 kg/m^2^ (obese) [*n* (%)]	278	94 (33.8)	287	92 (32.1)	
Gestational age at baseline (weeks)	271	14.92 ± 1.65	283	14.90 ± 1.71	0.881
Ethnicity [*n* (%)]					
White	271	257 (94.8)	280	255 (91.1)	0.120
Other	271	14 (5.2)	280	25 (8.9)	
Education, completed tertiary [*n* (%)]	262	153 (58.4)	277	190 (68.6)	**0.018**
Neighborhood deprivation index	278	5.48 (11.35)	287	6.39 (11.29)	0.342
Current Smoker [*n* (%)]	243	14 (5.8)	254	14 (5.5)	1.000
Primiparous [*n* (%)]	270	132 (48.9)	276	146 (52.9)	0.395
Supplement user [*n* (%)]					
Folic acid pre-pregnancy	208	106 (51.0)	218	118 (54.1)	0.577
Folic acid since conception	208	202 (97.1)	218	216 (99.1)	0.255
Other micronutrient supplements pre-pregnancy	208	55 (26.4)	218	54 (24.4)	0.885
Other micronutrients supplements since conception	208	58 (27.9)	218	64 (29.4)	0.509

a *Values are means ± SDs; unless specified as [n (%)]*.

b *P-values were calculated using independent samples t-tests for continuous variables and chi-squared tests for independence for categorical variables*.

### Dietary Outcomes

At baseline, there were no differences between intervention and control groups for mean daily GI and GL intakes. Compared with the control group, the intervention group had significantly lower dietary GI [mean difference (MD) −1.75; 95% CI −2.81, −0.71; *P* = 0.001] and GL [MD −14.84; 95% CI −22.27, −7.41; *P* < 0.001] at 28 weeks' gestation, adjusting for baseline (Table 2).

**Table 2:** Between-group comparison of energy and macronutrient outcomes at 28 weeks' gestation, adjusting for baseline data (*n* = 290)^a^.

**Nutrient**	**Intervention (*****n*** **=** **147)**		**Control (*****n*** **=** **143)**		**Mean difference (95% CI)**	***P***
	**Pre**	**Post**	**Pre**	**Post**		
Energy (kcal)	1883.9 ± 420.8	1707.6 ± 424.7	1874.9 ± 418.6	1876.3 ± 419.4	−173.34 (−257.48, −89.21)	**<0.001**
Energy (kJ)	7918.1 ± 1762.1	7177.8 ± 1780.6	7881.6 ± 1755.6	7888.8 ± 1762.0	−729.71 (−1084.11, −376.33)	**<0.001**
Protein (g)	79.4 ± 18.0	79.4 ± 20.5	79.4 ± 20.0	81.9 ± 19.5	−2.54 (−6.45, 1.370)	0.202
Protein (%TE)	17.1 ± 2.7	19.0 ± 3.7	17.1 ± 3.2	17.6 ± 3.1	1.34 (0.61, 2.06)	**<0.001**
Carbohydrates (g)	230.0 ± 55.3	207.1 ± 53.7	231.7 ± 56.3	226.6 ± 56.1	−18.74 (−30.33, −7.14)	**0.002**
Carbohydrate (%TE)	49.0 ± 6.1	48.7 ± 5.5	49.7 ± 6.6	48.4 ± 5.9	0.43 (−0.83, 1.69)	0.503
GI	59.4 ± 4.8	56.5 ± 4.5	58.2 ± 5.3	58.0 ± 4.9	−1.75 (−2.81, −0.71)	**0.001**
GL	136.4 ± 34.5	117.4 ± 33.3	135.1 ± 36.0	131.8 ± 36.1	−14.84 (−22.27, −7.41)	**<0.001**
Sugars (g)	86.5 ± 31.1	74.8 ± 27.3	87.8 ± 35.1	86.4 ± 31.4	−11.13 (−17.44, −4.82)	**0.001**
Sugars (%TE)	18.5 ± 5.2	17.6 ± 4.6	18.5 ± 5.6	18.4 ± 5.2	−0.80 (−1.86, 0.26)	0.137
Free Sugars (g)	39.5 ± 20.8	30.6 ± 17.9	38.3 ± 23.3	37.9 ± 21.2	−7.53 (−11.91, −3.15)	**0.001**
Free Sugars (%TE)	8.3 ± 3.6	7.1 ± 3.7	8.0 ± 4.2	8.0 ± 3.8	−0.98 (−1.81, −0.15)	**0.021**
Fiber (g)	20.2 ± 6.3	21.5 ± 6.4	20.5 ± 5.9	20.4 ± 5.8	1.23 (−0.00, 2.45)	0.050
Fat (g)	77.4 ± 24.3	67.4 ± 23.3	75.7 ± 23.5	77.0 ± 22.4	−10.39 (−14.93, −5.85)	**<0.001**
Total Fat (%TE)	36.6 ± 5.6	35.1 ± 5.4	36.0 ± 5.5	36.7 ± 4.5	−1.80 (−2.93, −0.67)	**0.002**
SFA (%TE)	4.7 ± 2.3	4.1 ± 2.1	4.6 ± 2.2	5.1 ± 2.3	−1.06 (−1.55, −0.58)	**<0.001**
PUFA (%TE)	5.6 ± 1.4	6.0 ± 1.5	5.7 ± 1.8	5.7 ± 1.5	0.31 (−0.05, 0.64)	0.091
MUFA (%TE)	13.3 ± 2.6	12.8 ± 2.5	12.9 ± 2.8	12.9 ± 2.7	−0.25 (−0.82, 0.32)	0.387
Sodium (mg)	2323.7 ± 681.3	2074.4 ± 670.6	2393.0 ± 778.0	2281.8 ± 713.3	−183.49 (−332.86, −34.11)	**0.016**
Potassium (mg)	2887.5 ± 675.4	2871.2 ± 658.7	2911.3 ± 754.7	2876.8 ± 678.2	5.06 (−130.70, 140.82)	0.942
Calcium (mg)	924.3 ± 297.1	883.7 ± 316.1	924.9 ± 338.6	965.6 ± 315.4	−81.63 (−148.09, −15.18)	**0.016**
Iron (mg)	11.0 ± 3.0	11.4 ± 3.0	11.8 ± 5.2	11.4 ± 3.2	−0.08 (−0.81, 0.65)	0.348
Iodine (μg)	136.0 ± 61.1	140.9 ± 58.0	142.4 ± 64.1	149.5 ± 61.5	−6.37 (−19.27, 6.54)	0.332
Vitamin D (μg)	3.1 ± 2.2	3.3 ± 2.1	3.4 ± 2.2	3.3 ± 2.7	0.10 (−0.44, 0.64)	0.715
Vitamin C (mg)	100.4 ± 56.7	95.1 ± 56.2	102.8 ± 57.0	96.5 ± 52.5	−0.58 (−12.45, 11.29)	0.923

a *All values are means ± SDs; 95% CIs in parentheses. P-values were calculated using analysis of covariance with adjustment for corresponding baseline variables*.

*Data in this table come from participants with complete food diaries at both 16 and 28 weeks' gestation. There were no-differences in proportion of energy under-reporters between groups. Analysis excluding energy under-reporters found significantly higher fiber intakes among the intervention group in late pregnancy vs. controls, adjusting for baseline (P = 0.004), no difference in percentage of energy from free sugars (P = 0.061), sodium (P = 0.149), or calcium (P = 0.074) in late pregnancy between the two groups, adjusting for baseline. % TE, percentage of total energy*.

*Bold value indicate significance level of <0.05*.

With respect to energy and macronutrient intakes, the intervention group had significantly lower post-intervention mean daily energy (kcal); carbohydrates (g); sugars (g); free sugars (g), (%TE); fat (g), (%TE); saturated fat (%TE); and higher protein (%TE) vs. controls, adjusting for baseline data (Table 2). In terms of micronutrients, significantly lower intakes of sodium (mg) and calcium (mg) were observed among women in the intervention group post-intervention, adjusting for baseline data (Table 2). However, in terms of micronutrient density, no differences were observed between the control and intervention for these two nutrients; sodium [control 289 mg/kJ vs. intervention 289 mg/1,000 kJ]; calcium [control 122 mg/kJ vs. intervention 123 mg/kJ].

At randomization, 11.3% of the cohort were identified as under-reporters of energy intake. The proportion did not differ between intervention and control groups [25 (11.2%) vs. 24 (11.4%); *P* = 1.0]. However, at 28 weeks', 14.7% were found to under-report energy intakes, with a higher proportion of those women in the intervention group [31 (19.5%) vs. 16 (9.9%); 153 *P* = 0.024].

### Stage-of-Change and Physical Activity Outcomes

**Figure 3:**
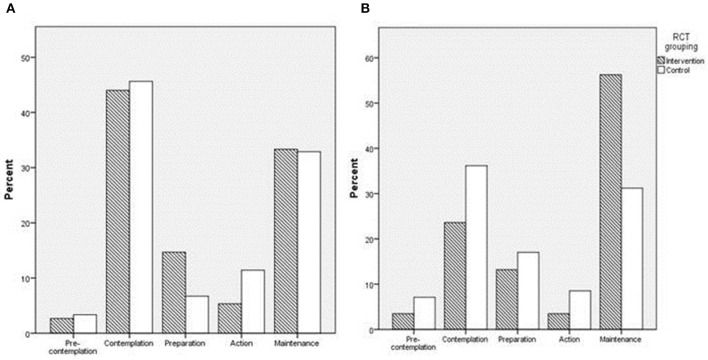
Bar charts representing the distribution of participant behavioral stage-of-change scores for physical activity in each group at **(A)**; 15.6 weeks', and **(B)**; 28 weeks' gestation.

There was no difference in behavior stage-of-change (Figure 3), self-reported physical activity levels or the rate of compliance to ACOG exercise recommendations between groups at baseline (Table 3). Pre-intervention, among the total group, most participants were at behavior stage-of-change 2 (contemplation), (44.0% intervention, 45.6% control), and stage 5 (maintenance) (33.0% intervention, 32.9% control) (*P* = 0.548) for perceived participation in physical activity (Figure 3). Post-intervention, the proportion of participants at stage 5 was higher in the intervention group compared to control (56.3 vs. 31.2%, *P* = 0.001) (Figure 3). Post-intervention, the intervention group also had higher total physical activity levels (MET-min/week), vs. controls, and higher moderate intensity activity (min/week), adjusting for pre-intervention data. However, the intervention did not influence the percentage of women meeting ACOG exercise recommendations (Table 3).

**Table 3:** Between-group comparison of self-reported physical activity measurements and compliance with ACOG recommendations at 28 weeks' gestation, adjusting for baseline data (*n* = 306)^a^^,^^b^.

	**Intervention (*****n*** **=** **153)**		**Control (*****n*** **=** **153)**				
	**Pre**	**Post**	**Pre**	**Post**	**Mean difference (95% CI)**	**Odds ratio (95% CI)**	***P***
Light activity (minutes per week)	71.5 ± 66.8	96.8 ± 71.9	76.1 ± 63.8	84.8 ± 66.3	13.3 (-3.1, 29.7)	–	0.111
Moderate activity (minutes per week)	59.5 ± 65.4	78.1 ± 69.5	55.3 ± 62.6	50.1 ± 60.1	26.5 (11.7, 41.3)	–	**0.001**
Vigorous activity (minutes per week)	11.8 ± 32.3	8.0 ± 25.1	9.9 ± 26.0	3.1 ± 13.9	4.0 (-5.9, 8.9)	–	0.087
MET-minutes per week	511.4 ± 459.2	609.8 ± 448.6	487.0 ± 397.5	463.9 ± 324.2	141.4 (62.9, 219.9)	–	**<0.001**
Meeting ACOG recommendations [*n* (%)]	33 (21.6%)	36 (24.7%)	28 (18.3%)	24 (15.9%)	–	1.63 (0.89, 2.99)	0.117

a *Values are means ± SDs; 95% CIs in parentheses, unless specified as [n (%)]*.

b *P-values were calculated using analysis of covariance (continuous data) and logistic regression (categorical data) with adjustment for corresponding baseline variables. Data in this table come from participants with complete questionnaires at both 16 and 28 weeks' gestation*.

*Light Activity (e.g., yoga, golf, easy walking, bowling, beginners aerobics, and light gardening)*.

*Moderate Activity (e.g., fast walking, tennis, badminton, easy swimming, easy cycling, popular and folk dancing, intermediate aerobics, and heavy gardening)*.

*Vigorous Activity (e.g., running, jogging, football, soccer, squash, basketball, vigorous swimming, vigorous long-distance cycling, and advanced aerobics)*.

*MET-minutes formula: 8·0 × vigorous activity + 4·0 × moderate activity + 3·3 × light activity*.

*Bold value indicate significance level of <0.05*.

### App Usage and Associated Diet and Physical Activity Outcomes

Of the 278 participants in the intervention arm, 76 never used the app, 5 only used the app on the day it was downloaded and 197 used the app on at least 2 days. Thus, there were *n* = 197 app-users, and *n* = 81 non-app-users. There were no differences in demographic characteristics between app-users and non-app-users at baseline (Supplemental Tables 1, 2).

**Figure 4:**
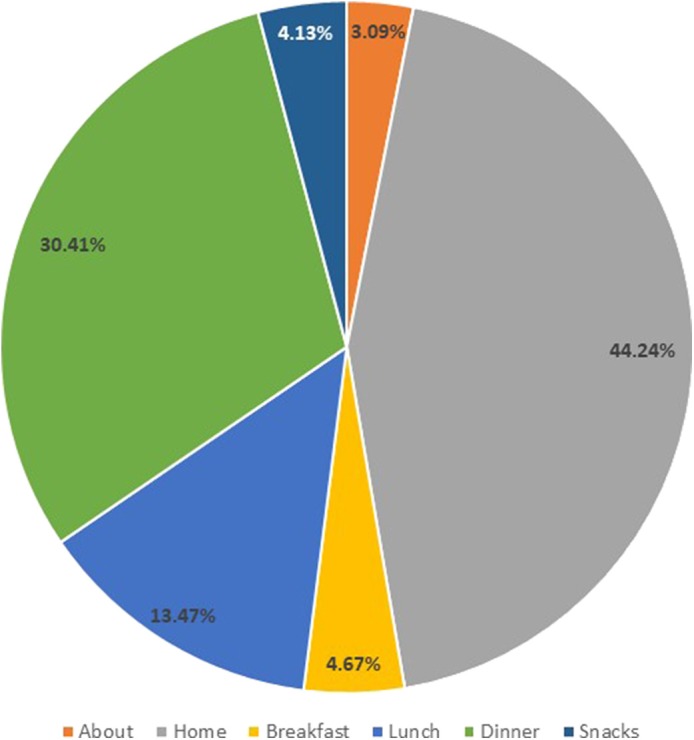
Frequency of use (%) for various areas of the study app.

Among app users, median (IQR) weeks of usage was 18.6 (12.9), total instances of app use were 22 (60) and use per week was 1.7 (2.6). The homepage was the most frequently accessed section of the app (Figure 4), followed by Dinner, Lunch, Breakfast, Snack, and “About” sections. There were no differences in nutrient intakes or physical activity levels between app-users and non-app-users at baseline. Mean GI and percentage of energy from free sugars were significantly lower among app-users vs. non-app-users at 28 weeks, but no differences in physical activity levels were observed (Table 4). Higher total instances of app usage were positively associated with fiber intake (g) at 28 weeks' gestation (B = 2.46, 95% CI = 0.304, 4.615). No other outcomes were associated with total instances of app use as a continuous variable.

**Table 4:** Comparison of dietary, physical activity and pregnancy outcomes between app-users and non-app-users.

	**App-user**		**Non-app-user**		
	**Mean**	**SD**	**Mean**	**SD**	***P*^**a**^**
**Dietary outcomes at 28 weeks' gestation**	***n*** **=** **126**		***n*** **=** **33**		
Energy (Kcal)	1714.2	411.7	1690.5	459.1	0.774
GI	56.1	4.5	58.0	4.4	**0.032**
GL	115.7	30.4	122.4	39.2	0.290
Carbohydrate (g)	206.1	51.7	209.4	56.1	0.743
%TE Carbohydrate	48.3	5.6	49.8	5.4	0.149
Sugars (g)	74.4	27.8	73.7	24.5	0.888
%TE sugars	17.4	4.7	17.7	4.6	0.723
Free sugars (g)	29.0	17.7	33.5	16.7	0.097
%TE free sugars	6.7	3.5	8.1	3.9	**0.041**
Fiber (g)	21.9	6.7	20.1	5.2	0.151
Protein (g)	80.4	18.9	76.3	17.3	0.254
%TE protein	19.1	3.6	18.4	3.4	0.337
Fat (g)	68.1	22.9	65.9	25.3	0.631
%TE Fat	35.3	5.6	34.5	5.0	0.440
%TE MUFA	12.8	2.6	12.7	2.5	0.866
%TE PUFA	6.0	1.6	5.9	1.6	0.560
%TE SFA	4.0	1.9	4.5	2.3	0.194
Sodium (mg)	2134.2	703.1	2038.9	648.6	0.483
Potassium (mg)	2874.9	671.5	2893.8	568.9	0.882
Calcium (mg)	914.4	328.8	811.2	221.5	0.091
Iron (mg)	11.5	2.9	11.0	3.2	0.344
Iodine (μg)	146.5	60.3	125.0	47.7	0.059
Vitamin D (μg)	3.5	2.4	3.0	1.8	0.303
Vitamin C (mg)	94.0	55.1	97.6	55.9	0.736
**Physical activity outcomes at 28 weeks' gestation**	***n*** **=** **136**			***n*** **=** **33**	
Mild physical activity (minutes/week)	96.6	72.6	97.5	73.6	0.953
Moderate physical activity (minutes/week)	76.5	68.0	82.2	72.7	0.687
Total physical activity (MET-minutes/week)	666.5	436.9	671.2	365.2	0.957
Met ACOG guidelines *n* (%)	78	(57.3)	19	(57.6)	0.981

a *Data are Mean, SD unless otherwise stated*.

*GI, glycaemic index; GL, glycaemic load; %TE, percentage total energy; ACOG, American Congress of Obstetricians and Gynecology; MET-minutes per week, metabolic equivalent of task minutes per week. Bold value indicate significance level of <0.05*.

## Discussion

This study demonstrates the effectiveness of a multifaceted antenatal lifestyle intervention, grounded in behavior-change theories, and supported by a smartphone app, to significantly improve dietary and exercise behaviors. The intervention reduced dietary GI, GL and improved some other nutrient intakes while also improving readiness to engage in physical activity behaviors, and higher reported physical activity levels. Greater engagement with the smartphone app was associated with some improvements in nutrient intake, but not physical activity.

The significant reduction in dietary GI and GL, and improvements in nutrient intakes, confirm previous findings from dietary and lifestyle interventions in pregnant cohorts, including women with obesity (2, 30). The reduction in GL in the current study was much greater than that observed in the ROLO study (30), but similar to that of the UPBEAT study (2), suggesting that a greater impact can be achieved with interventions that employ continuous modes of intervention delivery, either face to face (2), or via mHealth, and behavior-change techniques. In the current study, the change in carbohydrate intake, a reduction in grams of carbohydrate, as mainly total sugars and free (non-milk extrinsic) sugars, appears to drive the change in GI and GL. This reduction in carbohydrate intake did not affect the percentage of energy from total carbohydrates, however, the percentage of energy from total sugars and free sugars were reduced. Fiber intake also increased non-significantly in the intervention group. The improvement in nutrient intakes suggests better diet quality among women in the intervention group, a finding that is comparable with the improvement in Healthy Eating Index score in the LIMIT trial (11). The specific dietary modifications that resulted in a lower calcium intake in the intervention group vs. controls at 28 weeks' gestation requires further exploration, however, the calcium intake of both groups is comparable to previous studies of pregnant women (39).

Improvements in stage-of-change score support evidence that provision of physical activity advice during pregnancy increases the proportion of women in stages 4 (action) or 5 (maintenance) (36). These findings also support qualitative evidence that some women feel motivated to make healthy lifestyle changes in pregnancy, despite facing challenges in the implementation of such behavior changes (5). A meta-analysis shows previous interventions employing behavior-change theories and techniques effectively attenuated declining physical activity levels in pregnancy (3, 40). Although the PEARS trial did not significantly improve women's compliance to ACOG exercise guidelines, it did succeed in increasing moderate and total activity levels compared to the control group. These findings, in addition to comparable outcomes in similar cohorts (2, 41), suggest that behavioral interventions, supported by mHealth technologies, can improve exercise behaviors among women with a raised BMI.

The frequency of app usage reported in the current study is lower than anticipated. Daily use of the app was encouraged, but median use was once to twice per week and 22 instances in total. Despite this, app users had somewhat better dietary behaviors vs. non users. This could demonstrate the usefulness of the app in assisting women to choose and prepare healthier meals. It is not surprising that app usage was not associated with physical activity levels given that it was primarily focused on motivating women to adhere to a low GI diet. Low usage of mHealth supportive tools is not unique to this study. The recent app aspect of the SNAPP trial (18) and website “OptiMUM Nutrition” (19) found that approximately one third of pregnant women engaged with the app or repeat-visited a study website.

### Clinical Implications

National polices from around the world on the management of obesity in pregnancy suggest that women with a BMI >30 kg/m^2^ should be offered dietary advice and support to promote physical activity (42–45). Given the positive evidence to date regarding diet and lifestyle interventions in pregnancy and improved health outcomes for women with a BMI >25 kg/m^2^, it would be prudent to expand offerings of such interventions to women with overweight and obesity; for example, through lifestyle antenatal classes as part of routine clinical practice. The results of this trial also suggest the potential for mHealth, in the form of a smartphone app, to assist traditional lifestyle antenatal interventions to improve maternal health behaviors. Smartphones may capture a wide demographic (12) enabling health care professionals to engage with groups that are typically hard to reach. A concern for healthcare professionals is that the quality of freely available smartphone apps have consistently been shown to be poor (20, 21). This study app was developed by a multi-disciplinary team; dietitians, obstetricians and app developers, and all content was evidence-based. Implementing consistent and routine lifestyle support may be more feasible and cost-effective with the aid of a smartphone app to reinforce the message of the initial face-to-face meeting with a healthcare professional.

### Strengths and Limitations

A key strength of this study is that it was designed to be applied within routine clinical practice and minimize issues associated with traditional high-intensity interventions. This study also employed behavior-change theories, and specified BCTs to allow comparability of methods across similar studies and to ensure a theoretical basis for behavior-change was used (26, 27). Measures of physical activity were self-reported, which may not reflect objectively-measured levels as previously found among women with obesity in pregnancy (46). Self-reported dietary data used in this study also has limitations, and revealed a level of energy under-reporting in each group, which was higher among the intervention group at 28 weeks. Analyses excluding under-reporters mitigated the significant reduction in free sugars (%TE) and sodium among the intervention group, however, fiber intakes increased significantly, and no differences were observed in calcium intakes. Another limitation was the attrition rate in data collection of the food diaries. There is also risk of social desirability bias that can occur among participants in lifestyle interventions, and particularly those in the intervention group, such that reported dietary and physical activity behaviors may have been altered to reflect what is expected from the advice given to participants.

In conclusion, the findings of the current study build upon previous antenatal dietary and physical activity interventions. The results show that continuous lifestyle support, grounded in behavior-change theory, can assist pregnant women with a higher BMI to improve dietary intakes and physical activity. This trial highlights the potential for mHealth, specifically a smartphone app, to assist in delivering intervention content, and to further support women to engage in healthful lifestyle behaviors during pregnancy. Thus, the findings of this study are of clinical significance as similar approaches could be implemented within antenatal care as a routine service.

## Data Availability Statement

The datasets generated for this study are available on request to the corresponding author.

## Ethics Statement

The studies involving human participants were reviewed and approved by National Maternity Hospital Ethics Committee. The patients/participants provided their written informed consent to participate in this study.

## Author Contributions

FM, MK, and KL designed the research. KA, MK, OO'B, and EO'B conducted the research. KA and EO'B analyzed the data. All authors contributed to the manuscript. FM had primary responsibility for the final content of the manuscript.

### Conflict of Interest

The authors declare that the research was conducted in the absence of any commercial or financial relationships that could be construed as a potential conflict of interest.
