# Supplementary material for: Nutrition, Behavior Change and Physical Activity Outcomes From the PEARS RCT—An mHealth-Supported, Lifestyle Intervention Among Pregnant Women With Overweight and Obesity
Source: Front Endocrinol (Lausanne). 2020 Feb 4;10:938. doi: 10.3389/fendo.2019.00938 (PMC7010810; doi:10.3389/fendo.2019.00938)
Supplement: Supplementary file 1 [file Table_1.pdf]

Supplementary Table S1 Behaviour-change techniques employed based on the Coventry, Aberdeen & London – Refined (CALO-RE) Taxonomy, supporting behaviour-change theories, target intervention functions and behavioural constructs, and modes of delivery

Taxonomy Numbers Used	Behaviour Change Technique	Supports Behaviour Change Theory	Targets Intervention Function	Targets Behavioural Construct COM-B	Education Session/Follow up Appointments	Smartphone App	Follow Up Emails
1	Provide information on consequences of behaviour in general	Social Cognitive Theory, Theory of Planned Behaviour, Information-Motivation Behavioural Skills Model.	Education. Incentivisation.	Psychological capability. Automatic and Reflective Motivation.	Discuss both maternal and foetal health consequences of rapidly digested carbohydrates.	Provision of messages regarding healthy gestational weight gain.	Re-iteration of health consequences of poor dietary choices and lack of physical activity with regards to pregnancy and labour outcomes.
4	Provide normative information about others behaviour	Social Cognitive Theory, Information-Motivation Behavioural Skills Model.	Modelling.	Automatic Motivation.	Discuss examples of healthy dietary and physical activity habits other patients partake in.	Provision of messages regarding healthy habits in pregnancy.	X
5	Goal setting (behaviour)	Control Theory	Enablement.	Physical and Psychological Capability, Physical and Social Opportunity, Automatic Motivation.	SMART Goals set for increasing PA and eating more low GI carbohydrates. These goals are realistic and achievable.	X	X

Table S1 ***continued***

6	Goal setting (outcome)	Control Theory	Enablement.	Physical and Psychological Capability, Physical and Social Opportunity, Automatic Motivation.	Discuss gestational weight gain goal at education session.	X	X
8	Barrier identification/ Problem solving	Social cognitive Theory	Enablement.	Physical and Psychological Capability, Physical and Social Opportunity, Automatic Motivation.	Discuss most commonly occurring barriers individual participant changes when it comes to altering diet and physical activity. Help identify methods to overcome to suit participant lifestyle.	X	Further assistance/ suggestions provided on how to overcome newly identified barriers that occur throughout pregnancy.
10	Prompt review of behavioural goals	Control Theory	Persuasion.	Automatic and Reflective Motivation.	Review of individual low GI diet and physical activity prescription at follow up appointments.	X	Query how participants are doing with regards to previously discussed dietary and physical activity habits.
11	Prompt review of outcome goals	Control Theory	Persuasion.	Automatic and Reflective Motivation.	Review of GWG, glycaemic control	X	X

Table S1 *continued*

11					and anthropometry at follow up visits.		
12	Prompt reward contingent on effort or progress towards behaviour	Control Theory	Incentivisation.	Automatic and Reflective Motivation.	X	Positive encouragement towards healthy progress is provided through generalised messages.	Based on specific feedback from participant, praise and positive encouragement for progress towards behaviours is provided.
13	Prompt reward contingent on successful behaviour	Control Theory	Incentivisation.	Automatic and Reflective Motivation.	X	X	Based on specific feedback from participant, praise and positive encouragement for success with behaviours is provided.
15	Prompting generalization of target behaviour	Social Cognitive Theory	Education. Enablement. Environmental Restructuring.	Psychological Capability. Physical and Psychological Capability, Physical and Social Opportunity, Automatic Motivation.	Discuss how adoption of lower GI carbohydrates can be done at any meal and at any location. Discuss how physical activity is so	Messages to convey how healthy eating and physical activity are not confined to certain learned situations only.	Reinforce idea that changes to low GI diet and physical activity can be made in a variety of simple situations to allow full

Table S1 ***continued***

				Physical and Social Opportunity, Automatic Motivation.	diverse and simple to integrate.		integration of new habits into daily life.
17	Prompt self-monitoring of behavioural outcome	Control Theory	Enablement.	Physical and Psychological Capability, Physical and Social Opportunity, Automatic Motivation.	Discuss monitoring gestational weight gain at each antenatal visit during educational session and follow up appointments.	Reminder to keep track of gestational weight gain.	X
19	Provide feedback on performance	Control Theory	Persuasion	Automatic and Reflective Motivation.	At follow up appointments, positive feedback and encouragement is provided based on participant reporting of behaviours.	X	Positive feedback and encouragement provided based on participant reporting behaviours.
20	Provide information on where and when to perform the behaviour	Information-Motivation Behavioural Skills Model	Education. Environmental Restructuring.	Psychological capability. Physical and Social Opportunity, Automatic Motivation.	Discuss locations and times that best suit for engaging in physical activity and timing/spacing of meals throughout locations frequented in daily life.	Further messages on practical suggestions for performing exercise and cooking new foods.	Re-iterate information from original education session.

Table S1 ***continued***

21	Provide instruction on how to perform the behaviour	Information-Motivation Behavioural Skills Model	Training. Environmental Restructuring.	Physical and Psychological Capability. Physical and Social Opportunity, Automatic Motivation.	Discuss practical ways of including more physical activity: e.g. in intervals throughout day. Discuss how to measure out and cook low GI starchy carbohydrates.	Directions to prepare low GI meals. Directions to partake in safe exercises during pregnancy.	Discuss how to create low GI alternatives for seasonal meals.
23	Teach to use prompts/cues	Social Cognitive Theory	Persuasion.	Automatic and Reflective Motivation.	X	Provides daily prompts for improved health in pregnancy and healthy meal and exercise of the day.	Prompts reminder to use the app to further engage with original intervention content.
24	Environmental restructuring	Social Cognitive Theory	Environmental Restructuring.	Physical and Social Opportunity, Automatic Motivation.	Discuss healthy tips to incorporate peers, family and friends within physical activity routine and within changing dietary habits.	Provision of messages aimed at spouse, partner and family.	Provides healthy tips to incorporate peers, family and friends within physical activity routine and within changing dietary habits.
27	Use of follow up prompts	Social Cognitive Theory	Persuasion. Incentivisation.	Automatic and Reflective Motivation.	x	Provision of daily rotating messages based on core parts of original education session content.	Provision of fortnightly rotating messages based on core parts of

Table S1 ***continued***

27							original education session content.
29	Plan social support/Social change	Social Cognitive Theory	Environmental Restructuring.	Physical and Social Opportunity, Automatic Motivation.	Discuss integrating family and friends into physical activities and with reducing GI at meal times.	Provision of messages aiming to engage entire family with adopting new habits also.	Reinforce idea that spouse, partner, friends can provide can form a supporting structure to make healthy changes in pregnancy.
35	Relapse prevention/Coping planning	Social Cognitive Theory	Enablement. Coercion. Incentivisation.	Physical and Psychological Capability, Physical and Social Opportunity, Automatic Motivation. Physical and Social Opportunity, Automatic Motivation.	x	Helpful positive reminder to keep going when feeling low or incapable.	Details provided on how to manage when deviation from lifestyle change has occurred at points throughout intervention.
40	Stimulate anticipation of future rewards	Control Theory	Incentivisation.	Physical and Social Opportunity, Automatic Motivation.	Discuss benefits of improved energy levels, gestational weight gain and improved glycaemic control.	Prompted messages reinforcing the rewarding pregnancy benefits of eating well and exercising.	Reemphasise the benefits of continued exercise in pregnancy and importance in preparation for labour.

Supplementary Table S2 Additional behaviour-change techniques employed from The Behaviour Change Technique Taxonomy V1 and the modes of delivery

Behaviour Change Technique	Education Session/Follow up Appointments	Smartphone App	Follow Up Emails
Non-specific encouragement	Encouragement and enthusiasm to build self-efficacy.	Daily Motivational Messages for healthy pregnancy.	Further encouragement and enthusiasm consistent throughout remainder of pregnancy.
Behaviour Substitution	Discuss swapping higher GI foods for lower GI foods at main meals and snacks.	App provides healthy low GI alternatives to everyday meals and snacks.	When gestation advances, discuss suitable lower intensity exercises if difficulties arise with performing usual exercises.
Habit formation	Discuss good healthy eating habits such as always eating breakfast and eating frequently versus leaving long periods between eating occasions. Discuss achieving 30 minutes of exercise on most days through various practical methods.	Encourages physical activity and healthy eating in pregnancy and relates changes to positive pregnancy outcome.	Re -iterates making better physical activity habits and low GI food choices. Encourages daily use of the app to access readily available healthy meals.
Habit reversal	Discuss reducing consumption of sweet treats and white refined starchy carbohydrates. Discuss reducing portion sizes of starchy carbohydrates at meal times.	X	Discusses cutting back on weekly habits such as takeaways etc. and during certain times of the year e.g. Easter, Halloween, Christmas.
